# Supplementary material for: Manifestations, responses, and consequences of mistreatment of sick newborns and young infants and their parents in health facilities in Kenya
Source: PLoS One. 2022 Feb 22;17(2):e0262637. doi: 10.1371/journal.pone.0262637 (PMC8863216; doi:10.1371/journal.pone.0262637)
Supplement: Attachment — Submitted filename: Response to Reviewers.docx [file pone.0262637.s002.docx]

Avenue 5, 3rd Floor
Rose Avenue
Nairobi
Kenya

Tel: +254 020 513 4700

Email: [info.nairobi@popcouncil.org](mailto:info.nairobi@popcouncil.org)

# Editor

**PlosOne**

**12/10/2021**

**Dear Sir/Madam**

**Ref: Revision of the paper “Manifestations, responses, and consequences of mistreatment of sick newborns and young infants and their parents in health facilities in Kenya”**

**Comment from Reviewer #1: :** The paper is much clearer now and addresses better the complexity of this topic making the needed links with available literature. The authors may consider presenting the conclusions in the same order of thinking as the categories of mistreatment, for example starting from the need of addressing health system challenges, including availability of competent neonatal health care providers, then the issues of building better communication and responding to the developmental needs of infants and emotional needs of families, and finally creating supporting environments for care and bereavement.

**Response 1:** We thank the reviewer for these comments. The conclusion has been revised to reflect the suggested changes and now reads as presented below

*“This study outline types of mistreatment that were observed or reported for SYIs in five hospitals in Kenya and explores the responses and consequences of parents. Mistreatment for SYIs appears to be prevalent and linked to poor quality of care. To address mistreatment in this group of very young children, interventions that focus on building better communication, responding to the developmental needs of infants and emotional needs for the parents, strengthen the number of providers and their competencies in newborn care, as well as a supportive, enabling, and healthy environments, will lead to more respectful quality care for newborns and young infants*”

**Comment from Reviewer #2: Abstract:** Conclusions need to reflect the results of the study. Summarize them in simple words.
**Response 2:** This has been adjusted in line with review 1 and now reads

*“Mistreatment for SYIs is linked to poor quality of care. To address mistreatment in SYI, interventions that focus on building better communication, responding to the developmental needs of infants and emotional needs for parents, strengthen providers competencies in newborn care, as well as a supportive, enabling environments, will lead to more respectful quality care for newborns and young infants”*

**Comment 3: Methods**: I am sure this was not a cross sectional study. Please carefully write the methods section in the abstract as well as in the main methods section.

**Response 3**: This has been reworded in both the methods section of the study and the abstract. The corrected language in both sections removes the cross-sectional qualifier. It now reads

*“This was a qualitative formative study designed as part of larger implementation research to develop and test strategies for improving provision of nurturing care; promotion of family engagement; and communication and respect for care of newborns, infants, and very young children in resource-constrained facilities in Kenya”*

**Comment 4-1** Findings of the study. The analysis has been improved yet there needs to be effort for this analysis to meet the quality qualitative study expectations. A clear analytic approach is missing

**Response 4-1:** The analytic approach – thematic analysis – is described on page 8 in the “data processing and analysis section.” We have added some language around our mixed approach (deductive and inductive) to guide the reader more carefully through our adaptation of existing frameworks and application to our data

**Comment 4-2**. The findings are merely description of what participants said and believed. It needs to go beyond and identify the nuances within the data and then add interpretation. Follow some quality qualitative articles on your topic.

**Response 4-2**. We appreciate this comment made in several rounds of review. However, we feel that the approach we have taken is analytical and integrates categories known from literature in the field and then we demonstrate manifestations using data as examples. The integrative approach we have used is deliberate to illuminate examples of typologies we present. This we hope provides evidence via the mundane details we present.

**Comment 4-3**. The statement “An initial codebook of 19 themes and 100 sub-themes was developed and refined based on the process of open coding and progressive” is wrong. We cannot have that many themes or subthemes in qualitative research.

**Response 4-3**: We have removed the sub-theme categorization as it is irrelevant for this manuscript and retained the 19 themes which represent the main topic areas that contributed to the themes presented.

**Comment 4-4**. Identify only a few themes by merging categories and tell a complete connected story about how your theme answer your research question. And what was your research question needs to be explicitly identified.

**Response 4-4**: Our analysis was led both by deductive and inductive approaches and aligned to the existing typologies. Our story is then detailed using the typologies, but we go ahead and describe how these experiences affect caregivers of children and the reactions they exhibit. Our research intent was to describe perspectives of parents and providers, what constitutes mistreatment of SYIs, what drives provider behaviors, parents’ immediate responses to mistreatment of SYIs, and the consequences of these experiences on parents

**Comment 4-5.** We describe what constitutes mistreatment of SYIs, what providers say drives their behaviors, parents’ immediate responses to mistreatment of SYIs, and the consequences of these experiences on parents: it is I think the parents would perceive and describe their experiences of mistreatment not you who will describe it. Therefore, your objectives need to be reset.

**Response 4-5**: Appreciate the comment. We have reworded the objectives, by adding the clause “Drawing on perspectives of parents and providers,…we describe….” The use of “we” is an accepted way to draw the perspectives of the researchers into the interpretation of the data and study objectives – particularly relevant and helpful in qualitative work. What is meant here is, “The study describes…” We hope that with this change, the objectives are clearer.

**Comment 6**. Where you are referring to table 1, I believe you mean Fig. 1.

**Response 6**: This has been corrected

**Comment 7.** Discussion will follow the amendments based upon the suggestions on revising the themes.

**Response 7**: We have responded to the issues on themes and how we have structured them. We believe the analytical approach resonates with literature. We therefore did not make much adjustment to the discussions except editorial changes.

We believe the changes made above are sufficient and adequately responds to the issues raised

We look froward for your final decision

Timothy Abuya, PhD

On behalf of the team
